# Sexual Risk Behaviors and HIV Infection among Men Who Have Sex with Men and Women in China: Evidence from a Systematic Review and Meta-Analysis

**DOI:** 10.1155/2015/850132

**Published:** 2015-12-08

**Authors:** Hong-Yi Wang, Jun-Jie Xu, Hua-Chun Zou, Kathleen Heather Reilly, Christiana Meng Zhang, Ke Yun, Yong-Ze Li, Yong-Jun Jiang, Wen-Qing Geng, Hong Shang, Ning Wang

**Affiliations:** ^1^Key Laboratory of AIDS Immunology of National Health and Family Planning Commission, Department of Laboratory Medicine, The First Affiliated Hospital, China Medical University, Shenyang 110001, China; ^2^Collaborative Innovation Center for Diagnosis and Treatment of Infectious Diseases, Hangzhou 310000, China; ^3^Kirby Institute, University of New South Wales, Sydney, NSW 2052, Australia; ^4^National Center for AIDS/STD Control and Prevention, Chinese Center for Disease Control and Prevention, Beijing 102206, China; ^5^Georgetown University School of Medicine, 3900 Reservoir Road Northwest, Washington, DC 20007, USA

## Abstract

*Objectives*. To understand the current risk of HIV infection and transmission and further elucidate the underlying risk factors among men who have sex with men and women (MSMW) in China. *Methods*. Following PRISMA guidelines, we conducted a systematic review and meta-analysis of searching through Chinese and English available literature databases between January 2000 and June 2014 to identify articles. *Results*. Thirty-six articles (including 19,730 MSMW and 53,536 MSMO) met the selection criteria and the aggregated results found that MSMW have significantly higher HIV prevalence than MSMO (6.6% versus 5.4%, OR = 1.27, 95% CI = 1.01–1.58). A higher proportion of MSMW had commercial male partners in the past 6 months (18.3% versus 12.2%, OR = 1.56, 95% CI = 1.01–2.42). Additionally, substance use in the past 6 months was significantly more frequent among MSMW than MSMO (alcohol use: 27.1% versus 13.1%, OR = 2.53, 95% CI = 2.14–2.99; illicit drug use: 5.3% versus 2.5%, OR = 2.09, 95% CI = 1.48–2.95). *Conclusion*. A higher proportion of commercial sex and substance use among MSMW may be a potentially indicative factor for significantly higher HIV prevalence compared to MSMO. Targeted interventions should aim at increasing the frequency of HIV/STIs screening and preventing high risk commercial sex and substance use among MSMW to decrease their HIV transmission to the general population.

## 1. Introduction

Men who have sex with men (MSM) are considered as a group of high risk for HIV infection in many countries. According to The Joint United Nations Programme on HIV and AIDS (UNAIDS), the prevalence of HIV infection among MSM in capital cities from nearly 80 countries is on average 13 times higher than that of general populations in these countries [1]. This disparity is also prominent in China; a recent meta-analysis reported that almost 6.5% of MSM were living with HIV [2], which was more than 100 times higher than that in the general population (0.058%) [3]. The latest national report revealed that the proportion of newly diagnosed HIV cases due to male homosexual contact has increased from 12.2% in 2007 to 23.4% in 2014 [4, 5].

Homosexuality is still highly discriminated against in China, which partly results in bisexual behavior becoming one of the biggest obstacles in implementing HIV/AIDS prevention and control interventions among MSM [6, 7]. Additionally, marriage is a family obligation of Chinese traditional culture, which prompts over 80% of MSM eventually getting married to female to hide their real sexual identity and/or carry on family name [8, 9]. Only one in ten MSMW discloses homosexuality to their female partners [10, 11], and unprotected sexual behaviors with female sex partners were prevalent among MSMW [12], which may bridge HIV from the MSM population to the general population.

Previous studies have estimated peraction transmission probability of HIV during anal or vaginal sex among MSM [13, 14]. Clear understanding of the relative risk may help inform MSM of the potential risk which may in turn render them to avoid high risk sexual behaviors, especially among those who make condom use decisions based on penetrative sex type and role in anal sex (receptive or insertive anal sex) and self-reported HIV status of partner. However, until now little literature had vigorously compared the relative risk of HIV infection among MSMW, based on all available data in China.

Existing studies had contradictory findings on the relative risk of HIV infection comparing MSMW and MSMO in China. A recent published national cross-sectional survey of 47,231 MSM from 61 cities in China indicated that MSMW have a lower HIV prevalence than MSMO [15]. However, this conclusion is contradictory to a previous meta-analysis study, which found that MSMW in China have a significantly higher HIV prevalence than MSMO [16]. Until now, no new studies indicated whether or not Chinese MSMW really have higher risk of HIV infection than MSMO. An explicit assessment of the risk of HIV infection in MSMW populations is useful for understanding the dynamic of national HIV epidemic among MSM and developing targeted interventions. Additionally, many new researches on bisexual behavior of MSM have been published in recent years. As such, we also include the latest extensive literature in order to gain a more comprehensive and current understanding of national HIV epidemic among MSM in China. Besides, previous meta-analysis on bisexual behavior among MSM in China failed to explore the underlying differences in HIV-related behavioral risk factors between MSMW and MSMO, which are essential to contextualize behavioral interventions of high risk subpopulations among MSM in China. Therefore, we conducted this systematic review and meta-analysis to compare the disparities in HIV prevalence between MSMW and MSMO and examine behavioral factors underlying the disparities. Three research questions were addressed: (1) do MSMW in China have significantly higher HIV prevalence than MSMO; (2) what are the differences between risk behavioral factors of MSMW and MSMO in China; (3) do MSMW engage in more risk sexual behaviors in different proportions than MSMO that might help to explain the differences in the effect size for HIV prevalence?

## 2. Methods

### 2.1. Literature Search

This systematic review and meta-analysis adheres to the PRISMA guidelines [17, 18]; comprehensive literature search was conducted using the following databases for literature published between January 2000 and June 2014 to identify articles: PubMed, Web of Knowledge, Google Scholar, Chinese National Knowledge Infrastructure, VIP, and Wanfang Data. In addition, the reference lists of pertinent articles were examined for additional relevant studies. The combinations of search terms include “bisexual behavior,” “bisexual men,” “MSMW,” “men who have sex with men and women,” “BBM,” “behaviorally bisexual men,” “MSM,” “men who have sex with men,” “homosexual men,” “gay,” “MSMO,” “men who have sex with men only,” “HIV” and “risk behaviors,” “risk factor,” “unprotected sex,” and “condom use.”

### 2.2. Inclusion/Exclusion Criteria

Studies were included in the meta-analysis if they reported quantitative data on HIV prevalence and risky behaviors rate among both MSMW and MSMO. Studies were excluded if (1) they were duplicate reports; (2) they failed to report risky behaviors or HIV prevalence among both MSMW and MSMO; (3) HIV status was self-reported and not confirmed by test; (4) they did not mention the period of the recall window of risk behaviors; and (5) they were postintervention studies. For research data repeatedly be published in multiple articles, the most comprehensive article was included in the meta-analysis.

### 2.3. Quality Assessment

The quality of studies was assessed using the quality assessment checklist for observational studies (QATSO score), a validated quality assessment tool for HIV prevalence/risk behaviors among MSM [19]. Items were scored as 1, 0, and NA, which represents “yes,” “no,” and “not applicable,” respectively. The total score of each eligible study must have been above 33% (0% and 33%, 33% and 66%, and 67% and 100%, corresponded to “bad,” “satisfactory,” and “good quality,” resp.).

### 2.4. Data Extraction

Bisexuality was operationalized using a classification of MSMW or MSMO over any timeframe (behavior recall window) assessed by researchers. Data from eligible studies were extracted with the following information by two reviewers independently: (1) general information about each selected article was extracted (e.g., first author, publication year, study period, study location, method of recruitment, sampling method, and behavior recall window). (2) HIV infection: the prevalence of HIV infection. (3) Behavioral information: (1) condom use: unprotected anal intercourse (UAI) with males, unprotected receptive anal intercourse with males (URAI), and unprotected insertive anal intercourse (UIAI) with males and UAI with commercial male partner; (2) anal sex role: mainly insertive anal intercourse (IAI), mainly receptive anal intercourse (RAI), and both of the two roles; (3) male partner: multiple (≥2) male partners have casual male partners and have commercial male partner (include purchasing sex from male sex workers (money boys and MBs) or selling sex to males); (4) substance use: alcohol use and ever illicit drug use (including opiate, cocaine, amphetamine-type stimulants (ATS), cannabis, and hallucinogens [20]).

### 2.5. Statistical Analysis

To assess the differences in HIV infection and related risky behaviors between MSMW and MSMO, the random/fixed effect models were used to compute the pooled effect rates and odds ratios (OR) (i.e., prevalence and OR of HIV infection and proportion and OR of condom use, anal sex role, sex partners, and substance use) and relevant 95% confidence intervals (CI). Statistical heterogeneity was qualitatively tested using Cochran's *Q* statistic (*p* < 0.10 indicates significant heterogeneity) and quantified by the *I* ^2^ index (*I* ^2^ < 25%, low heterogeneity; *I* ^2^ = 25–50%, moderate heterogeneity; *I* ^2^ > 75%, high heterogeneity). If significant homogeneity was detected (*I* ^2^ < 75.0, *p* ≥ 0.10), fixed effect models were employed to calculate the pooled effect rates and ORs; otherwise random effects models were employed [21].

There were many factors that may have affected the homogeneity between studies, such as sample characteristics and methodological differences. Therefore, we performed subgroup analyses to explore the potential sources of between-study heterogeneity on (1) study region: Southwest China, East China, Northeast, North China, and multiple regions; (2) data collection period: prior to 2009, 2009 and later, and unidentified; (3) data collection method: interviewer-administered, self-administered, and unidentified.

A sensitivity analysis was conducted by omitting each study one at a time to assess the influence of each study on the overall estimate. To investigate publication bias, we utilized Egger's regression test and examined the symmetry of funnel plots for each comparative meta-analytic domain. All the statistical analyses were done using STATA V11.2.

## 3. Results

### 3.1. Study Selection

**Figure 1:**
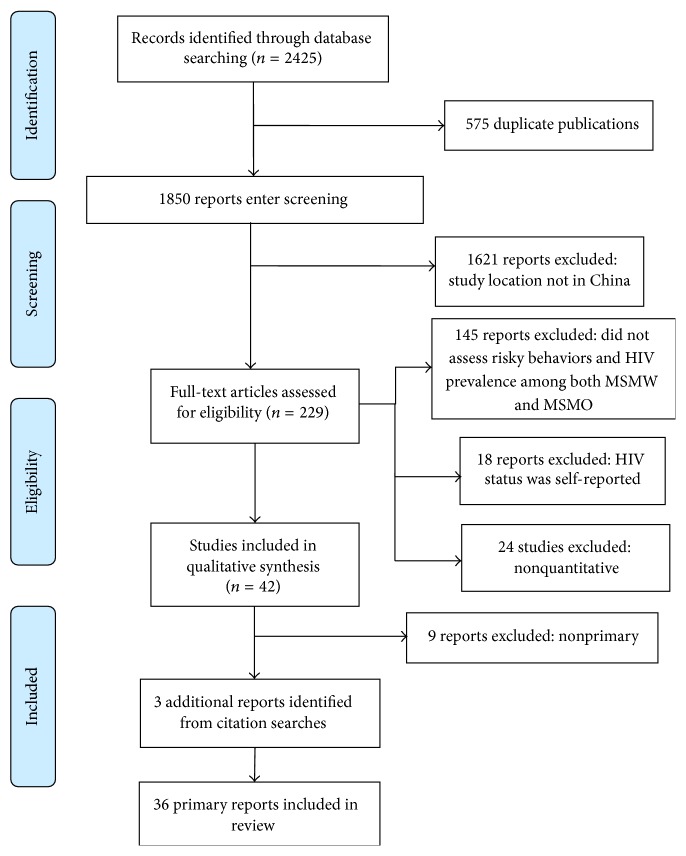
Flow diagram of studies included in analysis.

As shown in Figure 1, a total of 2425 relevant articles were identified, of which 42 articles entered further screening, and 36 articles (16 published in English and 20 in Chinese, including 19,730 MSMW and 53,536 MSMO) were finally included in our systematic review and meta-analysis (Figure 1) [15, 22–56]. The characteristics of these included studies are summarized in Table 1.

### 3.2. Quantitative Data Synthesis

#### 3.2.1. HIV Prevalence Analysis and Comparison

**Figure 2:**
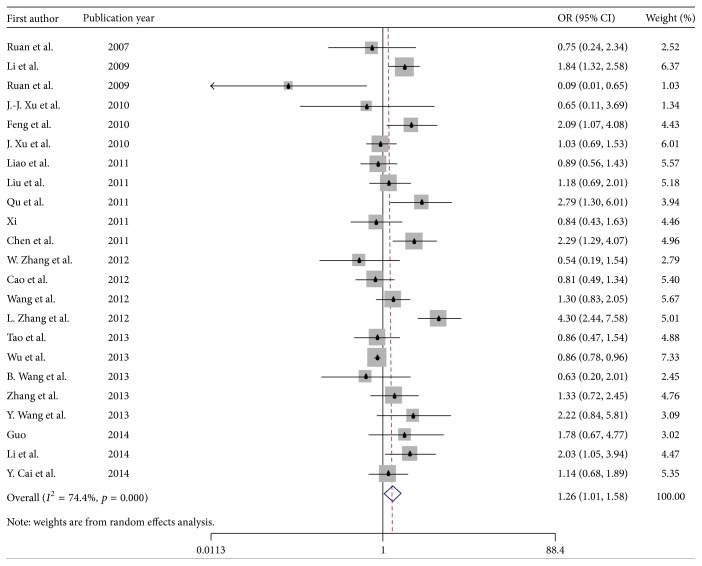
Forest plot of OR pool estimates of HIV infection among MSMW, compared to MSMO.

As seen in Table 2, across 23 samples, a significantly higher prevalence of HIV was found among MSMW compared with MSMO (6.6% versus 5.4%, OR = 1.27, 95% CI = 1.01–1.58) (Figure 2). Subgroup analysis by region showed that in the Southwest China, the difference in HIV prevalence between MSMW and MSMO was the highest (14.8% versus 6.7%, OR = 2.43, 95% CI = 1.58–3.72), followed by Northeast China (6.5% versus 5.7%, OR = 1.27, 95% CI = 0.89–1.82), the East China (2.7% versus 5.7%, OR = 0.67, 95% CI = 0.32–1.42), North China (5.7% versus 4.1%, OR = 1.32, 95% CI = 0.89–1.94), and other regions (6.3% versus 6.0%, OR = 1.03, 95% CI = 0.78–1.36). For studies that collected data in 2009 and later, the gap in HIV prevalence between MSMW and MSMO was larger than in other periods (8.3% versus 5.6%, OR = 1.59, 95% CI = 1.08–2.33). The prevalence of HIV for studies recruiting participants through RDS and all other sampling methods was 6.9% versus 6.3%, OR = 1.16, 95% CI = 0.65–2.09 and was 6.7% versus 5.2%, OR = 1.30, 95% CI = 1.05–1.61, respectively.

#### 3.2.2. Possible Explanatory Behavioral Factors for Significant Difference in HIV Infection Risk

As seen in Table 3, for anal sex role with male, MSMW were more likely to have insertive anal intercourse (IAI) and less likely to have receptive anal intercourse (RAI) than MSMO (IAI: 53.2% versus 41.1%, OR = 1.74, 95% CI = 1.26–2.42; RAI: 23.7% versus 38.7%, OR = 0.42, 95% CI = 0.28–0.64). No significant differences were found in condom use between MSMW and MSMO (UAI: 56.7% versus 57.8%, OR = 1.19, 95% CI = 0.99–1.26; UIAI: 63.9% versus 58.3%, OR = 1.19, 95% CI = 0.89–1.60; URAI: 45.9% versus 53.3%, OR = 0.73, 95% CI = 0.50–1.06; UAI with commercial male partner: 53.2% versus 41.1%, OR = 1.07, 95% CI = 0.76–1.50). In comparison with sex partners in the past 6 months, MSMW were significantly more likely to engage in commercial sex than MSMO (18.3% versus 12.2%, OR = 1.56, 95% CI = 1.01–2.42). Yet a marginally significant higher proportion of MSMW have multiple male partners (57% versus 51.9%, OR = 1.19, 95% CI = 0.85–1.67, *p* = 0.090) and casual male partners (65.3% versus 61.3%, OR = 1.16, 95% CI = 0.85–1.58) in the past 6 months. Additionally, substance use was significantly more frequent among MSMW than MSMO (alcohol use in the past 6 months: 27.1% versus 13.1%, OR = 2.53, 95% CI = 2.14–2.99; illicit drug use in the past 6 months: 5.3% versus 2.5%, OR = 2.09, 95% CI = 1.48–2.95).

### 3.3. Sensitivity Analysis and Publication Bias

The pooled rates and ORs were not significantly affected in the sensitivity analysis, suggesting that the results were robust. The funnel plot (dots nearly symmetrically distributed) and Egger's test (all *p* values for Egger's test > 0.05) reflected that there was no evidence of publication bias in any comparison model (data not provided).

## 4. Discussion

To our knowledge, this is the first systematic review to compare both HIV prevalence and various risk behaviors between Chinese MSMW and MSMO. It provides an insight into the latest HIV epidemic of MSMW and specific HIV transmission risk from MSM to general population and proposes a crucial suggestion for China's health department to make more effective prevention strategy and policy in the future. Building on our earlier research, we integrated the latest extensive 31 additional published pieces of literature and further validated that Chinese MSMW have a significantly higher HIV prevalence than MSMO, which is similar to our previous conducted meta-analysis results [16]. Remarkably, our present systematic review further clarified risk behavioral differences among MSMW and MSMO in China, which have not yet been disclosed before.

The aggregated ORs among MSMW were 1.3 times more likely to be HIV-positive than MSMO, which is quite different from a meta-analysis of American MSM suggesting that HIV prevalence among MSMW is somewhat lower than MSMO (OR = 0.41, 95% CI: 0.31–0.54) [57]. These divergences may be attributed to relatively lower rates of risky behavior of MSMW than MSMO in the United States, such as fewer UAI and URAI exposures. On the contrary, higher rates of commercial sex and substance use were found in Chinese MSMW in present systematic review.

The HIV prevalence among MSMW varies according to geographic differences: the extraordinarily high HIV prevalence of MSMW in the Southwest compared to other regions in China (Southwest China: 12.9%; Northeast China: 6.5%; North China: 5.1%; East China: 2.7%). Due to the fact that Southwest China includes several high HIV prevalence areas in China such as Guangxi and Yunnan [3, 58], MSMW in this region engaged in similar sexual risk behaviors may have higher HIV prevalence than in other regions of China. So targeted measures should be taken, which consider the risk profile of MSMW in high HIV prevalence areas of China in order to curb the spread of HIV.

Moreover, our findings further showed that Chinese MSMW have a high rate of commercial sex in the last 6 months. As MSMW feared exposing their homosexual orientation to their female spouse or partner, some may choose to buy sex from MBs [59]. On the other hand, MBs in China often sell sex to both males and females to increase their income [24]. MBs have become one of the emerging high risk subgroups of MSM communities in recent years [60–62], so higher rate of commercial sex in MSMW was associated with HIV transmission. The current systematic review also estimated that MSMW were 2.1 and 3.0 times more likely to engage in alcohol use than MSMO. Moreover, MSMW more likely use illicit drugs in this study. Such social environments may lead MSMW to hide their sexual orientation by unwillingly engaging in heterosexual relationships, given that they may seek out substance use (alcohol/illicit drugs) in an attempt to modulate pressure from reality [22]. As substance use may relax safer sex norms and increase unprotected anal sex, a higher substance use rate in MSMW may increase the risk of HIV transmission [63–65]. Our findings are consistent with other studies that MSMW prefer insertive anal sex role [28, 30, 31, 34, 57, 66–68], but we failed to find significant differences in condom use with male partners between MSMW and MSMO; hence, insertive anal sex role reducing the risk of HIV transmission may be offset by significant higher rate of commercial sex and using alcohol/illicit drugs.

The findings yielded in this study have some important implications for national HIV prevention and care planning and intervention development. The results suggest that MSMW have composed a significant special subproportion of the population of MSM who are both vulnerable to HIV infection from other higher risk MSM and also in high risk of bridging HIV epidemic to their female partners. However, there are currently no HIV prevention targeted interventions policies for MSMW in China. Given this challenge, we should develop specific prevention strategies targeting MSMW from the following three aspects. First, strategies should focus on increasing HIV/STIs screening programs for MSMW subpopulations to help them to learn their HIV infection status. We could promote and implement VCT or STIs clinic-based interventions to increase the frequency of HIV/STIs screening among MSMW through more frequent calling or sending of text message to remind them with their clinic visits [69]. Second, we should take measure of MSMW on preventing high risk commercial sex and substance use; meanwhile, we should enhance their consciousness of protecting themselves as well as their partners (both male and female). Third, given high risk of HIV infection among MSMW, MSMW's family is also a potential source of HIV discordant couples in China [8, 70–72]. In recent years a number of international studies have shown that the early initiation of antiretroviral therapy (ART) could reduce rates of sexual transmission of HIV-1 and clinical events [73–75], in China; early ART initiation was also reported that may reduce HIV transmission in discordant couples [76]. Hence, early ART initiation for HIV-positive MSMW should also be a good candidate strategy to improve the quality of MSMW's lives and decrease their HIV transmission risk in discordant couples. Additionally, it is essential to actively advocate for the respect and social equality of people with bisexual and homosexual orientations and reduce the social discrimination and family pressures for MSM.

This systematic review and meta-analysis has several important limitations. First, the paucity of existing research did not allow for subgroup analyses of HIV prevalence by race/ethnicity and other demographic differences both within MSMW and compared to MSMO. Second, though we attempted to be as inclusive as possible, our available database searches may have excluded relevant studies from this systematic review and meta-analysis. Third, the majority of included articles of this study recruited participants in urban locations, so rural representation was limited. Fourth, for lack of data from Northwest and other areas in China, it is difficult to reflect the comprehensive national HIV epidemic. Fifth, the results of this study suggest that a higher proportion of commercial sex and substance use among MSMW may be a potentially indicative factor for significantly higher HIV prevalence compared to MSMO, but whether commercial sex and/or substance use significantly moderated HIV prevalence differences between MSMW and MSMO should also be verified in future research.

More emphasis needs to be placed on Chinese MSMW in order to develop more targeted prevention measures for these potentially hidden MSM. Strategies targeting MSMW focused on increasing the frequency of HIV/STIs screening, preventing high risk commercial sex and substance use, encouraging safe sex practices, and actively carrying out early antiretroviral therapy for HIV-positive MSMW; meanwhile, actively advocating for the respect and social equality of people with bisexual and homosexual orientations and reducing the social discrimination and family pressures for MSM could help to slow the spread of HIV/AIDS.

## Figures and Tables

**Table 1:** Study characteristics of 36 samples included in meta-analysis.

Author	Publication year	Location	Study periods	Setting	Sampling method	Survey method	Recall window (months)	QACO score (%)
Tang [55]	2007	Harbin	N/A^#^	Multiple recruiting methods	Snowball sampling	Interviewer-administered	6	40
Chen [51]	2008	Fuzhou	2008	MSM venue	Multiple sampling methods	Interviewer-administered	6	60
Ouyang et al. [50]	2008	Chongqing	2006	Multiple recruiting methods	Snowball sampling	Interviewer-administered	6	33
Bai and Feng [43]	2010	Liuzhou	2008	Multiple recruiting methods	N/A	Interviewer-administered	6	75
Han et al. [35]	2012	China	2008	N/A	N/A	N/A	6	60
She et al. [34]	2012	4 cites	2008	N/A	Snowball sampling	Interviewer-administered	6	60
Li et al. [48]	2009	Chongqing	2006	MSM venue	Snowball sampling	Interviewer-administered	6	40
Zhang et al. [31]	2012	Wuhan	N/A	MSM venue	Stratified cluster sampling	Interviewer-administered	6	80
Ruan et al. [47]	2009	Jinan	2008	N/A	RDS^##^	Interviewer-administered	6	60
Chow et al. [29]	2013	Changsha,Tianjin	2011	MSM venue	Convenience sampling	Interviewer-administered	6	75
Cai et al. [24]	2014	Shenzhen	2010	Community-based	RDS	CASI^*∗*^	6	80
Liao et al. [41]	2011	4 cities in Shandong Province	2008-2009	Multiple recruiting methods	Multiple sampling methods	Interviewer-administered	6	80
Wang et al. [52]	2007	Harbin	2006	MSM venue	N/A	Interviewer-administered	6	60
Ruan et al. [53]	2007	Beijing	2005	N/A	Multiple sampling methods	Interviewer-administered	6	80
Xu et al. [44]	2010	Liaoning	2006	Recruiting by NGO^*∗∗*^	N/A	Interviewer-administered	12	60
Lau et al. [49]	2008	Kunming	2003–2005	Multiple recruiting methods	Convenience sampling	Interviewer-administered	6	60
Cao et al. [37]	2012	4 cities	2008	Recruiting by NGO	Snowball sampling	Interviewer-administered	6	80
Liao et al. [22]	2014	4 cities in Shandong Province	2011	Multiple recruiting methods	N/A	Interviewer-administered	6	70
Tao et al. [28]	2013	Beijing	2010-2011	Multiple recruiting methods	Multiple sampling methods	Interviewer-administered	12	60
Wu et al. [15]	2013	61 cities	2008-2009	N/A	RDS + Snowball sampling	Interviewer-administered	6	90
Guo et al. [36]	2012	Beijing	2009	Multiple recruiting methods	N/A	Self-administered	6	70
Wang et al. [33]	2012	Harbin	2006–2010	Community-based	Snowball sampling	Self-administered	12	65
Cao et al. [30]	2013	Nanchang	2011	Multiple recruiting methods	Multiple sampling methods	Interviewer-administered	1 week	60
Li et al. [56]	2014	Beijing	2009–2011	N/A	RDS	CASI	12	80
Feng et al. [46]	2010	Chengdu	N/A	Multiple recruiting methods	Snowball sampling	Interviewer-administered	6	50
Wang et al. [27]	2013	Beijing	2008-2009	Multiple recruiting methods	Multiple sampling methods	Interviewer-administered	6	80
Zhang et al. [32]	2012	Chongqing	2009	N/A	RDS	CASI	6	80
Zhang et al. [25]	2013	Harbin	2011	Recruiting by NGO	N/A	Interviewer-administered	12	60
Cai et al. [23]	2014	Shenzhen	2009–2012	N/A	Snowball sampling + RDS	Interviewer-administered	6	75
Liu et al. [40]	2011	Beijing	N/A	MSM venue	Snowball sampling	Interviewer-administered	12	50
Wang et al. [26]	2013	Mianyang	2011-2012	N/A	Snowball sampling	Interviewer-administered	6	55
Qu et al. [39]	2011	Inner Mongolia	2010	Community-based	Snowball sampling	Interviewer-administered	6	60
Xu et al. [45]	2010	4 cities	N/A	N/A	Snowball sampling + RDS	Interviewer-administered	6	70
Xi [38]	2011	Hangzhou	2009-2010	MSM venue	Snowball sampling	Interviewer-administered	6	50
Chen et al. [42]	2011	Lanzhou	2006–2010	MSM venue	Snowball sampling	Interviewer-administered	6	50
Li [54]	2007	Beijing	2005	Multiple recruiting methods	Multiple sampling methods	Interviewer-administered	6	70

^#^N/A: not available, ^##^RDS: respondent-driven sampling, ^*∗*^CASI: computer-assisted self-administered interview, and ^*∗∗*^NGO: Non-Governmental Organizations.

**Table 2:** The odds and prevalence of HIV among MSMW and MSMO by study and design characteristics.

Category	Subgroup	Comparison group	Number of studies	Prevalence estimate (%)	Heterogeneity		OR and 95% CI	*p* value	Heterogeneity	
				and 95% CI	*I* ^2^ (%)	*p* value			*I* ^2^ (%)	*p* value
Overall		MSMW	23	6.6 (5.3, 7.8)	89.2	<0.001	1.27 (1.01, 1.58)	0.018	74.4	<0.001
		MSMO	23	5.4 (4.7, 6.2)	83.9	<0.001				

Regions	Southwest China	MSMW	4	14.8 (9.3, 20.3)	79.5	0.002	2.43 (1.58, 3.72)	0.004	53.3	0.093
		MSMO	4	6.7 (4.0, 9.5)	83.2	<0.001				
	East China	MSMW	3	2.7 (0.4, 4.9)	87.4	<0.001	0.67 (0.32, 1.42)	0.236	61.2	0.076
		MSMO	3	5.7 (1.7, 9.6)	89.5	<0.001				
	Northeast China	MSMW	3	6.5 (2.3, 10.7)	76.1	0.015	1.27 (0.89, 1.82)	0.101	0.0	0.740
		MSMO	3	5.7 (2.3, 9.2)	73.4	0.023				
	North China	MSMW	7	5.2 (4.0, 6.5)	20.0	0.277	1.32 (0.89, 1.94)	0.088	43.0	0.104
		MSMO	7	4.1 (3.1, 5.1)	48.8	0.069				
	Several cities from different regions or those that cannot be classified into the above four categories	MSMW	6	6.3 (4.5, 8.2)	82.3	<0.001	1.03 (0.78, 1.36)	0.304	61.6	0.023
		MSMO	6	6.0 (4.8, 7.2)	72.0	0.003				

Sampling method	RDS	MSMW	5	6.9 (3.8, 10.0)	95.5	<0.001	1.16 (0.65, 2.09)	0.218	88.9	<0.001
		MSMO	5	6.3 (5.0, 7.6)	85.4	<0.001				
	All other nonprobability sampling	MSMW	18	6.7 (5.1, 8.3)	84.8	<0.001	1.30 (1.05, 1.61)	0.037	47.4	0.014
		MSMO	18	5.2 (4.2, 6.3)	83.3	<0.001				

Data collection period	Before 2009	MSMW	7	4.9 (2.8, 6.9)	94.4	<0.001	0.91 (0.63, 1.32)	0.103	75.1	<0.001
		MSMO	7	5.8 (4.2, 7.4)	92.6	<0.001				
	2009 and later	MSMW	10	8.3 (5.9, 10.7)	71.8	<0.001	1.59 (1.08, 2.33)	<0.001	65.3	0.002
		MSMO	10	5.6 (4.1, 7.1)	77.4	<0.001				
	Not reported or cannot be classified into the above two categories	MSMW	6	6.5 (4.6, 8.5)	75.8	0.001	1.33 (0.97, 1.84)	0.376	48.8	0.082
		MSMO	6	5.0 (3.8, 6.2)	61.9	0.022				

Data collection method	Interviewer-administered	MSMW	19	6.5 (5.1, 7.9)	90.5	<0.001	1.21 (0.94, 1.56)	0.245	77.1	<0.001
		MSMO	19	5.6 (4.8, 6.5)	84.3	<0.001				
	Self-administered	MSMW	2	5.4 (2.8, 8.1)	23.5	0.253	1.37 (0.91, 2.08)	0.211	0.0	0.567
		MSMO	2	3.9 (2.9, 4.8)	0.0	0.449				
	Not reported or cannot be classified into the above two categories	MSMW	2	8.5 (3.4, 13.6)	72.7	0.056	1.58 (1.01, 2.50)	0.030	0.0	0.354
		MSMO	2	5.7 (0.1, 11.2)	89.2	0.002				

**Table 3:** The odds and proportion of HIV-related risky behaviors among MSMW and MSMO.

Behaviors	Number of studies	Comparison group	Prevalence estimate (%)	Heterogeneity		OR and 95% CI	*p* value	Heterogeneity	
			and 95% CI	*I* ^2^ (%)	*p* value			*I* ^2^ (%)	*p* value
Condom use in the past 6 months									
UAI with male	9	MSMW	56.7 (46.2, 67.2)	94.6	<0.001	1.19 (0.99, 1.26)	0.123	40.7	0.096
		MSMO	57.8 (50.4, 65.2)	95.0	<0.001				
UIAI with male	3	MSMW	63.9 (57.8, 70.0)	0	0.857	1.19 (0.89, 1.60)	0.101	23.2	0.272
		MSMO	58.3 (46.2, 70.4)	94.3	<0.001				
URAI with male	3	MSMW	45.9 (13.4, 78.4)	95.1	<0.001	0.73 (0.50, 1.06)	0.317	35.9	0.210
		MSMO	53.3 (32.9, 73.8)	98.0	<0.001				
UAI with commercial male partner	3	MSMW	53.2 (22.8, 83.5)	90.1	<0.001	1.07 (0.76, 1.50)	0.785	35.7	0.211
		MSMO	41.1 (10.2, 72.6)	94.7	<0.001				

Anal sexual role									
Mainly insertive	8	MSMW	53.2 (37.5, 68.9)	97.9	<0.001	1.74 (1.26, 2.42)	0.004	81.6	<0.001
		MSMO	41.1 (29.5, 52.8)	98.4	<0.001				
Mainly receptive	8	MSMW	23.7 (13.9, 33.4)	96.8	<0.001	0.42 (0.28, 0.64)	0.013	83.9	<0.001
		MSMO	38.7 (23.0, 54.5)	99.2	<0.001				
Both	5	MSMW	59.4 (53.9, 64.9)	96.8	<0.001	0.92 (0.69, 1.24)	0.256	62.2	0.032
		MSMO	35.8 (20.3, 51.3)	98.7	<0.001				

Sex partner in the past 6 months									
Multiple (⩾2) male partners	5	MSMW	57.0 (40.8, 73.2)	95.3	<0.001	1.19 (0.85, 1.67)	0.090	69.6	0.010
		MSMO	51.9 (42.8, 60.9)	94.7	<0.001				
Having casual male partners	4	MSMW	65.3 (56.8, 73.7)	78.6	0.003	1.16 (0.85, 1.58)	0.141	56.6	0.075
		MSMO	61.6 (53.1, 70.1)	94.5	<0.001				
Having commercial male partners	10	MSMW	18.3 (11.6, 25.1)	95.3	<0.001	1.56 (1.01, 2.42)	0.023	86.8	<0.001
		MSMO	12.2 (8.1, 16.4)	96.8	<0.001				

Substance use in the past 6 months									
Alcohol use	6	MSMW	27.1 (15.7, 38.2)	96.6	<0.001	2.53 (2.14, 2.99)	<0.001	33.5	0.185
		MSMO	13.1 (7.6, 18.6)	96.7	<0.001				
Illicit drug use	3	MSMW	5.3 (4.1, 6.5)	0	0.558	2.09 (1.48, 2.95)	<0.001	0	0.435
		MSMO	2.5 (1.9, 3.1)	0	0.536				
